# Identification of Critical Amino Acids in the IgE Epitopes of Ric c 1 and Ric c 3 and the Application of Glutamic Acid as an IgE Blocker

**DOI:** 10.1371/journal.pone.0021455

**Published:** 2011-06-27

**Authors:** Natalia Deus-de-Oliveira, Shayany P. Felix, Camila Carrielo-Gama, Keysson V. Fernandes, Renato Augusto DaMatta, Olga L. T. Machado

**Affiliations:** 1 Laboratório de Química e Função de Proteínas e Peptídeos/Centro de Biociências e Biotecnologia, Universidade Estadual do Norte Fluminense - Darcy Ribeiro, Campos dos Goytacazes, Rio de Janeiro, Brazil; 2 Laboratório de Biologia Celular e Tecidual/Centro de Biociências e Biotecnologia, Universidade Estadual do Norte Fluminense - Darcy Ribeiro, Campos dos Goytacazes, Rio de Janeiro, Brazil; University Paris Sud, France

## Abstract

**Background:**

The allergenicity of *Ricinus communis* L. (castor bean, Euphorbiaceae) is associated with components of its seeds and pollen. Castor bean allergy has been described not only in laboratory workers, but also in personnel working in oil processing mills, fertilizer retail, the upholstery industry and other industrial fields. In the present study, we describe the critical amino acids in the IgE-binding epitopes in Ric c 1 and Ric c 3, two major allergens of *R. communis*. In addition, we also investigate the cross-reactivity between castor bean and some air and food allergen extracts commonly used in allergy diagnosis.

**Methodology/Principal Findings:**

The IgE reactivity of human sera from atopic patients was screened by immune-dot blot against castor bean allergens. Allergenic activity was evaluated *in vitro* using a rat mast cell activation assay and by ELISA. Cross-reactivity was observed between castor bean allergens and extracts from shrimp, fish, gluten, wheat, soybean, peanut, corn, house dust, tobacco and airborne fungal allergens. We observed that treatment of rat and human sera (from atopic patients) with glutamic acid reduced the IgE-epitope interaction.

**Conclusions/Significance:**

The identification of glutamic acid residues with critical roles in IgE-binding to Ric c 3 and Ric c 1 support the potential use of free amino acids in allergy treatment.

## Introduction

Castor bean (*Ricinus communis* L.) contains approximately 50% oil, which has special characteristics such as a high viscosity, a high stability under heat and pressure, a low freezing point, and the ability to form waxy substances upon chemical treatment. As 0energy demands increase and fossil fuels are limited, the development of alternative renewable fuels becomes imperative. Interest in biodiesel has been increasing owing to its environmental benefits and renewability [1]–[3]. As castor beans are a good biofuel source [3], castor bean cultivation is likely to increase, posing a risk of exposure to pollen allergens [4]–[6]. In previous studies, major castor bean allergens were identified [7]–[11]. We have recently reported the identification of IgE-binding epitopes of castor bean seed allergens, defining four continuous epitopes in Ric c 3 and two in Ric c 1 [12].

In the present study we identify critical amino acids for IgE binding and investigate cross-reactivity with allergens typically used for allergy diagnosis. Initially, we utilized the glutamic acid-specific Woodward's Reagent K, WRK, (*N*-ethyl-5-phenylisoxazolium-3′-sulfonate) [13] to demonstrate the importance of the glutamic acid carboxylic group in the Ric c 1 and Ric c 3 epitopes in IgE binding. In addition, pre-incubation of atopic patient serum with free amino acids (glutamic acid) blocked IgE epitope interaction sites. We also have observed cross-reactivity between castor bean and airborne and food allergens, which was also reduced by incubation with dicarboxylic amino acids.

## Methods

### Plant material and 2S albumin purification

Castor bean (*R. communis L*., cultivar IAC-226) seeds were obtained from the Instituto Agronômico de Campinas, São Paulo/Brazil. The 2S albumin fractions were isolated and characterized by SDS-PAGE and immunoblotting experiments as described previously [9]. The 2S albumin isoforms, Ric c 1 and Ric c 3 as well as their polypeptide chains were isolated as described by [12].

### Animals and antiserum

Isogenic female R/A Tor rats, generally high producers of IgE, were obtained from the animal facility of the Universidade Federal Fluminense, Niteroi, RJ – Brazil, and all experimental procedures were approved by the animal research ethics committee of this University (Proc. CEUA-UENF/112). Immunizations with the 2S albumin pool (Ric c 1 plus Ric c 3) and serum separation were conducted as previously described [12].

### Extraction of IgG by affinity chromatography

IgG and IgE from human and rat sera were separated by affinity chromatography using protein G-Sepharose beads in batch mode with 1.5 mL polypropylene tubes (Sigma protocols). Thirty micro litres of total serum of immunized R/A Tor rat was mixed with the 100 µL of resin and with 50 µL of equilibration buffer. This mixture was incubated for 16 hours under stirring at 4°C. The mixture was centrifuged and the supernatant denoted FE (fraction likely enriched in IgE) was removed and stored at 4°C. After removal of the supernatant, the resin was washed ten times with 100 µL of equilibration buffer to remove the remaining uncoupled material. The immunoglobulin coupled to protein G-Sepharose was eluted using 100 µL of a 0.5 M acetic acid. The acidic solution was immediately neutralized by the addition of 1 M Tris-HCl pH 9.0. After washing and centrifugation, the eluted fraction, denoted FG (fraction likely enriched in IgG), was isolated. The presence of IgG was investigated in both FE and FG by dot blotting. Mast cell degranulation assays in the presence of FG or FE were also performed to identify the immunoglobulin involved in mast cell activation.

### Affinity Dot Blotting

Human serum analysis: sera from five patients who had total IgE values of 106 to 1527 KU/L were evaluated for their ability to recognize 2S albumin from *R. communis* by dot blotting. After the first evaluation, patient serum with high intensity recognition of castor bean allergens was used in subsequent assays.

For dot blot assays, 2S albumin or synthetic peptide (10 µg in 10 µL/dot) was spotted onto a nitrocellulose membrane and allowed to dry. The nitrocellulose membrane was incubated with total serum (1∶50) or affinity supernatant or eluted fractions, FE and FG, from human or rat serum. Secondary anti-rat IgG or anti-human biotin IgE (0.5 mg/mL) (both diluted 1∶2000) was then added to the membrane for 1 hour. Two hours later, IgG was detected using a rabbit anti-rat IgG- HRP conjugate (1∶2500). For IgE detection, the membrane was subsequently incubated with streptavidin-biotinylated HRP complex for 1 h. The colour of all probes was developed with a substrate mixture: 5 mg of DAB in 4.9 mL of water, 300 µL of 0.1 M imidazole, 100 µL of Tris-HCl 2 M buffer (pH 7.5) and 5 µL of 30% H_2_O_2_.

### Rat peritoneal mast cells

Wistar rats were obtained from the animal facility of the Universidade Estadual do Norte Fluminense Darcy Ribeiro (UENF). All experimental procedures were approved by the animal research ethics board of the UENF (Proc. CEUA-UENF/112). Rats (weighing ∼250 g) were euthanized with CO_2_ and a peritoneal wash was performed by injection of 20 mL of DMEM (Dulbecco's Modified Eagle Medium) containing 12 U/mL of heparin. The abdomen was gently massaged for approximately 90 s. The peritoneal cavity was carefully opened and the fluid containing peritoneal cells was aspirated with a Pasteur pipette. Thereafter, the cells were transferred to Petri plates and incubated for 30 min at 37°C. Two-thirds of the supernatant was aspirated and discarded. The mast cell-rich supernatant (1.8×10^5^ mast cells/mL) was separated into 100 µL aliquots and kept at room temperature.

### Mast cell degranulation assays and cross-reactivity

Rat peritoneal mast cells (100 µL) were incubated with pre-immune serum (control) and activated for 60 min at 37°C using 2S albumin polyclonal anti-rat IgE (2S alb AR IgE). After sensitization with 2S alb AR IgE, cells were washed twice with DMEM. Each experiment was carried out in the presence or absence of the synthetic peptides and a 2S albumin pool (100 ng). After incubation with antibodies and potential allergens (synthetic peptides, 2S albumin), histamine contents were determined (see below) and the cells (in 10 µL) were stained for 15 min with 10 µL of a solution containing 0.1% toluidine blue, 10% formaldehyde and 1% acetic acid, pH 2.8, allowing the visualization of degranulated mast cells. Granulated and degranulated mast cells were counted under a light microscope using the 40 X objective in a Neubauer chamber.

In order to investigate cross-reactivity between 2S albumin from *R. communis* and allergens used for allergy diagnosis, mast cells previously sensitized by 2S alb AR IgE were incubated with airborne and food allergens from FDA Allergenic (FDA-PRICKIT- Lot 04AK00001 and FDA-FOODKIT lot 04AK00004).

The airborne allergens that were tested were from dust, airborne fungi, wool, dust mites, grass, flowers, tobacco, cat epithelium, dog epithelium, horse epithelium, bovine epithelium, epithelia mix, feathers, cotton, horsehair, grass, chamomile, cockroaches, pyrethrum, silk, and cattails. The food allergens were from poultry, shrimp, beef, pork, egg, egg yolk, milk, fish, rice, gluten, corn, wheat, soybean, pepper, pineapple, cocoa, cashew, peanuts, orange, lemon, tomato and strawberry. Protein content measurements of the allergen solutions from FDA allergenic were performed using the Bradford method using egg albumin as a standard. Stock solutions containing 10 µg/mL were prepared and each test used 10 µL of these allergen solutions. Mast cell activation was evaluated by taking the percentage of granulated and degranulated cells as described previously and by the quantification of histamine released.

### Histamine quantification

After incubation with antibodies and allergens, the cell suspensions (100 µL) were centrifuged at 170 xg for 10 min. An aliquot of the supernatant (20 µL) was removed for histamine detection. The released histamine was submitted to ion exchange chromatography and quantified using a previously described protocol [12].

### Identification of amino acids involved in IgE binding

The peptides previously identified as IgE-binding epitopes in Ric c 1 or Ric c 3 allergens by Felix et al. [12] were synthesized utilizing the solid phase method. The synthetic peptide sequences are displayed in Table 1. All six peptides (P0 to P5) contained two or more dicarboxylic amino acid residues. In order to investigate which amino acid was able to bind to sensitized mast cells, two strategies were employed: chemical modification of synthetic peptides using Woodward's Reagent K (WRK) and treatment of IgE-mast cells with free amino acids as a potential IgE blocker.

**Table 1:** Identification of IgE epitopes from Ric c 1 and Ric c 3.

Sample/Amino acid sequences of the peptide	Degranulationin the presence of total serum	Degranulation in the presence of IgE (affinity FE)	Degranulation in the presence of IgG (affinity FG)
**Mast cell isolation**(negative control)	30 (+1,0)	30 (+1,0)	30 (+1,0)
**2S albumin**(positive control)	70 (+1,0)	78,0 (+1,6)	38,8 (+1,7)
**P0/** **E**GLRQAI**E**QQQSQGQ	74 (+0,9)	75,0 (+0,5)	33,1 (+1,8)
**P1/** **E**SKG**E**R**E**GSSSQQCR	64 (+0,4)	69,9 (+1,7)	38,4 (+0,1)
**P2/**Q**E**VQRKDLSSC**E**RYL	70 (+0,5)	67,9 (+1,1)	39,0 (+0,7)
**P3/**Q**E**QQNLRQCQ**E**YIK	65 (+0,7)	65,7 (+0,3)	39,7 (+0,3)
**P4/**D**E**CQC**E**AIKYIA**E**DQ	67 (+0,5)	67,2 (+0,6)	38,4 (+0,9)
**P5/**LHG**EE**S**E**RVAQRAG**E**	54 (+0,2)	64,4 (+0,6)	36,0 (+0,8)

### Modification with Woodward's Reagent K (WRK)

WRK is a specific reagent for modifying dicarboxylic amino acids [3]. Samples (500 µg of peptides or 1000 µg of the "pool" of 2S albumin) were treated with the WRK solution (250 mM in 0.001 M HCl). Fifty micro litres of samples and 50 µL of WRK solution were kept at 35°C for 3 hours. Peptide re-purification and chemical modification were evaluated by reverse-phase HPLC and mass spectrometry. The allergenicity of treated peptides or 2S albumin was analysed by the mast cell degranulation assay as described above.

### Evaluation of chemical modifications using reverse-phase high performance liquid chromatography

To verify the modifications on the proteins after chemical treatments, the samples, synthetic peptides and 2S albumin were subjected to reverse-phase liquid chromatography on a C18 column (Sephasil peptide C18 5 µST 4.6/250, flow rate of 0.7 mL min^−1^ using 0.1% trifluoroacetic acid (TFA) as solvent A and 80% of acetonitrile containing 0.1% TFA as solvent B.) using a Shimadzu apparatus. The elution profile was monitored by on-line measurement of the absorbance at 220 nm. Fractions containing the major peaks were dried in a speed Vac system. The UV spectrum was obtained using a photo-diode detector.

### Blockage of IgE

Mast cells sensitized with IgE were treated with 100 ng of aspartic acid, glutamic acid or a mixture containing 100 ng of each other amino acids present in the synthetic peptide (Gly, Leu, Arg, Gln, Ala, Ile, Ser for P_0_; Ser, Lys, Gly, Arg, Gln, Cys for P_1_; Gln, Val, Arg, Lys, Leu, Ser, Cys, Tyr, for P_2_; Gln, Asn, Leu, Arg, Cys, Tyr, Ile, Lys for P_3_; Cys, Gln, Ala, Ile, Lys, Tyr, for P_4_ and Leu, His, Gly, Ser, Arg, Val, Ala, Gln, for P_5_). Amino acids were dissolved in DEMEN. After free amino acid treatment, cells were incubated with 100 ng of each synthetic peptide or allergenic fractions (Ric c 1 or Ric c 3). Mast cell degranulation was evaluated as described, and the percentages of degranulation and histamine release were compared with those triggered by synthetic peptides or by allergenic isoforms in the absence of free amino acid treatment.

### Quantification of human IgE using ELISA

During the development of this project, there was no contact with atopic patients. Sera were used after performing security tests, and this project was not submitted to the human ethics committee because patients were not identified. Specific IgE levels were tested by ELISA with 20 µg/well of castor bean allergen. Plates were incubated with serum from one atopic patient diluted 1∶50. Bound IgE antibodies were detected with biotin-anti-human IgE (1∶2000 diluted) followed by streptavidin-peroxidase conjugate (1∶1000 diluted) (Pharmingen). The peroxidase reaction was developed and the optical density (OD) was measured at 492 nm. Each value represents the mean of two assays. OD Values <0.1 were considered negative.

### ELISA inhibition and mast cell activation assay for analysis of glutamic acid as an IgE blocker

The ability of glutamic acid to inhibit the binding of allergic patients' IgE to Ric c 1 and Ric c 3 was assessed by ELISA inhibition and mast cell activation assays. Briefly, serum from an atopic patient (427 KU/L- 1∶5 diluted) and Wistar rats were preincubated for 1 h with glutamic acid (end concentration 10 µg/mL). The ELISA or mast cell degranulation assays were performed as previously described. The percentage of IgE binding inhibition achieved by the pre-incubation treatment was calculated as follows: percentage of IgE binding  = 100–(OD_I_/OD_T_ X 100); OD_I_ represents the absorbance after incubation of glutamic acid-treated human serum; and OD_T_ represents the absorbance of untreated human sera. Mast cell granulation or degranulation was counted by optical microscopy and histamine release was quantified as described above.

## Results

### Fractionation of serum immunoglobulins by affinity chromatography

**Figure 1:**
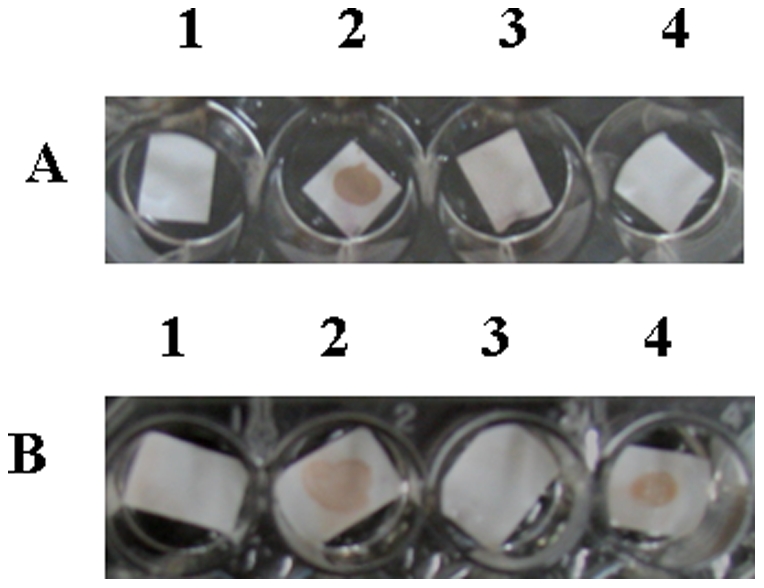
Analysis of rat affinity chromatography fractions FE and FG by dot blotting using a rabbit anti-rat IgG- HRP conjugate. A1 and B1) negative controls: water plus rat serum anti-2S albumin; A2 and B2) positive controls: 2S albumin plus rat serum anti-2S albumin; A3 and B3) affinity buffer plus anti-rat IgG; A4) 2S albumin incubated with FE plus anti-rat IgG; B4) 2S albumin incubated with FG plus anti-rat IgG.

Initially, the serum immunoglobulins (IgG and IgE) were separated by affinity chromatography. Two fractions denoted FE (supernatant fraction) and FG (eluted fraction) were obtained and the presence of IgG was investigated in both fractions by dot blotting. Figure 1 shows the lack of IgG in the supernatant fraction FE (spot A4), while IgG was detected in eluted fraction (spot B4), confirming that this immunoglobulin was removed by affinity chromatography. The supernatant fraction FE was used during mast cell degranulation assays.

The synthetic peptides present in Ric c 1 and Ric c 3 structures, which were recognized by the pool of sera and induced mast cell degranulation as described by Felix et al. [12], were tested in the presence of the FG or FE fractions. Table 1 shows the sequences of these peptides and indicates that mast cell degranulation is mediated only by IgE. The values of mast cell degranulation in the presence of total serum or FE fractions were similar, ranging from 64 to 78%, whereas degranulation mediated by FG was disregarded as it was similar to the negative control (approximately 30%).

### Importance of carboxylic groups in IgE binding

We found that the peptides identified as IgE epitopes had at least two glutamic acid residues (Table 1, residues marked in bold). To verify that these residues are essential for IgE binding, we examined mast cells incubated with IgE in the presence of synthetic peptides modified by Woodward's Reagent K. The first step of the reaction with WRK is the formation of an irreversible ketoketenimine that can be identified by a peak of absorbance at 340 nm [14]. The modification of the synthetic peptides by the WRK reagent was confirmed by specific spectra and by the increase of the peptide molecular weights by 254 Dalton or multiples of this value, indicating the inclusion of at least one *N*-ethyl-5-phenylisoxazolium-3′-sulfonate group (data not shown) by each glutamic residue. WRK-modified peptides were employed in mast cell degranulation assays and the results are shown in Table 2. After WRK treatment, peptide-induced mast cell degranulation was severely reduced to levels (30–39%) similar to that observed for the negative controls (30–39%). These data indicate the importance of carboxylic groups on IgE binding and in the mast cell degranulation.

**Table 2:** Percent of mast cell degranulation induced by Ricinus communis-derived synthetic peptides.

Peptide number	Mast cell degranulation by untreated peptide	Mast cell degranulation by WRK-treated peptide
**Control cells**	30±3	30±3
**P0**	74±4	37±1
**P1**	62±2	37±2
**P2v**	65±4	30±1
**P3**	63±2	33±2
**P4**	65±4	37±1
**P5**	53±2	39±1

### Dicarboxylic amino acids inhibit mast cell degranulation

Mast cells preloaded with IgE from rat serum were pre-incubated with free amino acids and exposed to a 2S albumin pool or IgE epitopes (synthetic peptides). The rationale of our experiment is illustrated in Figure S1.

The process of isolation of mast cells from rat peritoneal cavities induces ∼30% degranulation and 2% histamine release; thus, these values were set as a baseline for the negative controls. Castor bean 2S albumin induced 70% mast cell degranulation and more than 40% histamine release when incubated in the presence of IgE (Table 3).

**Table 3:** Critical role of dicarboxylic amino acid in mast cell degranulation induced by 2S albumin: percent of mast cell degranulation in the presence of the total serum that had been incubated with or without pooled amino acids.

Sample/Degranulation in the presence of total serum	% of Histamine released	Degranulation in the presence of total serum incubated with pooled amino acids	Degranulation in the presence of total serum incubated with Asp	Degranulation in the presence of total serum incubated with Glu	% of Histamine released after incubation with Glu
30 (+1.0 ) (negative control)	2	30.0 (±1.0)	30.0 (±1.0)	30.0 (±1.0)	2
70 (+1.0) (Positive control)	64	69.5 (±0.5)	36.8 (±.47)	29.7 (±0.1)	6
P0/74(+0.9)	80	63.0 (±0.7)	40.6 (±0.6)	37.6 (±0.6)	52
P1/64(+0.4)	58	67.1 (±0.7)	39.6 (±1.8)	37.0 (±1.9)	6
P2/70(+0.5)	64	63.5 (±0.4)	40.4 (±0.4)	30.3 (±1.6)	12
P3/65 +0.7)	58	65.2 (±0.7)	40.3 (±0.2)	33.8 (±1.7)	4
P4/67(+0.5)	60	63.8 (±0.8)	39.2 (±0.2)	36.7 (±0.6)	8
P5/54 +0.2)	56	63.4 (±0.9)	41.5 (±0.3)	38.8 (±1.4)	4

When mast cells were incubated with untreated serum or with serum treated with neutral or basic amino acids, degranulation triggered by allergens could be observed (Table 3, column 4). However, dicarboxylic amino acids protected IgE-bound mast cells from degranulation as described in Table 3 columns 5 to 7, implying that binding of IgE to castor bean 2S albumin was specifically blocked by glutamic or aspartic acid.

Glutamic acid inhibits IgE binding to castor bean allergens.

**Figure 2:**
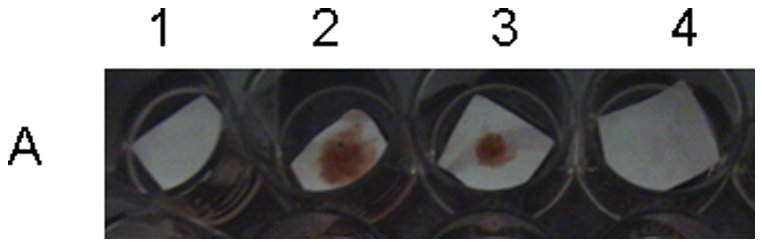
Analysis of human serum and affinity chromatography fractions, FE and FG by dot blotting using anti-human biotin IgE/streptavidin-biotinylated-HRP. A1) negative control: buffer plus human (patient 3) total serum; A2) 2S albumin plus human (patient 3) total serum; A3) 2S albumin incubated with affinity chromatography fraction, FE; A4) 2S albumin incubated with affinity chromatography fraction, FG.

**Figure 3:**
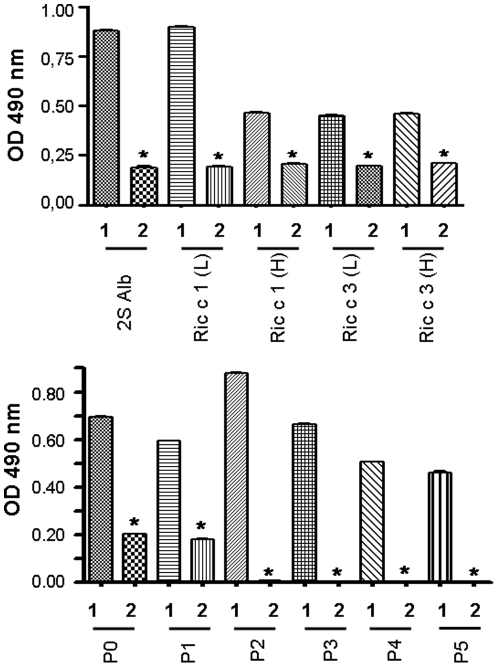
Glutamic acid reduces human IgE binding. ELISA binding assays of 2S albumin pool, 2S albumin light chain (L), heavy chain (H) and synthetic peptides with IgE from human serum without treatment (1) and with human serum treated with glutamic acid (2). Mean values from 5 experiments ± SD are presented. Statistical analysis was performed using one-way ANOVA followed by Turkey's post-test. * p<0.001.

Initially, the presence of anti-2S albumin IgE specific for castor bean allergen in sera from five atopic patients was evaluated by dot blotting, and the results are presented on Table 4. The serum from patient number 3 (total IgE- 427 KU/L) was the most positive for 2S albumin IgE and was selected for further study. This serum was fractionated by affinity chromatography and the presence of IgE was confirmed by dot blotting analysis to be only in the FE supernatant fraction as presented in Figure 2 (spot 3). We also used ELISA to examine the binding of this atopic patient serum to the 2S albumin pool and their isolated light and heavy chains and synthetic peptides P0 to P5. Higher affinity was observed for the 2S albumin pool, P2 and for the Ric c 1 light chain (Figure 3); however, binding was also observed for all tested samples.

**Table 4:** Detection of IgE anti 2S albumin in Human serum by dot blotting.

Patient	SerumTotal IgE (KU/L)	Dot Blotting
Control cells	1527	-
1	266	+
2	427	+++
3	143	-
4	108	-
5	1527	-

The possible blocking effect of glutamic acid was evaluated by ELISA experiments with serum from an allergic patient, and the results are presented in Figure 3. Pre-incubation of serum with glutamic acid inhibited by 78% the IgE binding when 2S albumin pool or Ric c 1 light chain was tested and inhibited by 56% when Ric c 1 heavy chain or both chains from Ric c 3 were used. The IgE binding to P0 and P1 were inhibited by 70% and was completely inhibited to other IgE epitopes, P2, P4 and P5 (Figure 3), confirming the results obtained with sensitized mast cells (Table 3).

### Cross-reactivity between castor bean 2S albumin and airborne/food allergens

The results obtained with mast cells previously sensitized with castor bean allergens and triggered by cross-reactivity with commercial allergens are shown in Table 5. Considering positive reactions to be above 50% degranulation, significant IgE reactivity to food extracts from shrimp, fish, gluten, wheat, soybean and peanut were observed. Cross-reactivity was also observed with inhalable allergens as house dust, tobacco and airborne fungus (Table 5). Mast cell degranulation for other foods tested as strawberry, birds, beef, pork, egg white, egg yolk, tomato, milk, rice, pineapple, cocoa, cashew, orange and lemon and for other aeroallergens as mites, wool, grass-plot, flowers, cat dander, dog epithelium, equine epithelium, bovine epithelium, mixed epithelium, feather mix, cotton, mane, grass, silk, flax, cockroach and cattail were below 35% (data not shown).

**Table 5:** Percentage of degranulation triggered by cross-reaction between 2S albumin from *Ricinus communis* L. and food or aero allergens.

Samples	Degranulation (%) in the absence of total serum(± S.D.)	Degranulation (%) in the presence of total serum(± S.D.)	Degranulation (%) after blockage with Glu (± S.D.)	Degranulation (%) after blockage with Asp (± S.D.)
Negative control	27.6 (±1.2)	30.0 (±1.0)	30.0 (±1.0)	30.0 (±1.0)
2S Albumin	32.6 (±1.3)	70.0 (±1.0)	29.7 (±0.1)	36.8 (±0.4)
Shrimp	34.0 (±0.7)	53.8 (±0.8)	34.0 (±1.0)	41.6 (±1.0)
Fish	38.8 (±1.0)	57.6 (±0.3)	34.9 (±0.2)	40.6 (±0.3)
Gluten	38.6 (±1.0)	56.3 (±0.5)	35.5 (±0.3)	40.6 (±0.3)
Wheat	38.8 (±0.2)	56.0 (±0.9)	36.8 (±0.5)	41.6 (±0.2)
Soybean	27.4 (±0.4)	62.5 (±0.4)	32.7 (±0.3)	39.7 (±0.3)
Peanut	30.2 (±0.3)	51.4 (±0.3)	35.2 (±0.5)	40.6 (±0.4)
Corn	34.4 (±1.0)	54.4 (±1.5)	34.1 (±0.3)	40.6 (±0.5)
House dust	36.7 (±0.2)	51.4 (±0.4)	33.9 (±0.7)	40.0 (±0.2)
Tobacco	34.8 (±0.2)	55.0 (±0.9)	34.6 (±0.1)	43.2 (±0.5)
Air fungus	48.7 (±0.7)	51.9 (±0.9)	32.3 (±0.4)	40.0 (±0.6)

Dicarboxylic amino acids (glutamic or aspartic acid) were used as IgE blocker.

### Glutamic acid protects against cross-reactivity

As observed in Table 5 (columns 4 and 5), pre-absorption of serum with glutamic acid resulted in inhibition of IgE reactivity to both inhalable and food allergens, indicating that the carboxyl group of these amino acids could be important in IgE-epitope interactions.

## Discussion

The use of castor bean oil in biodiesel production will result in the increased planting of castor beans. Castor bean allergens are present not only in the seeds but also in the pollen of this plant [5], [15]. Thus, identifying the allergenic epitopes and reactive amino acids are critical steps toward the development of strategies to reduce IgE binding, which will undoubtedly help protect rural workers or those in direct contact with the seeds.

To determine which amino acids are involved in IgE binding, the chemical modification of synthetic peptides by WRK was employed. Yost and Anderson [16] suggested the importance of a carboxyl group in the NAD-glycohydrolase activity of a *Bungarus fasciatus venom* protein as this enzyme is rapidly inactivated by WRK treatment. To confirm the importance of carboxylic groups in IgE binding, we have performed blocking assays using free amino acids (glutamic and aspartic acids) (Table 3). The dicarboxylic amino acids protected mast cell activation by IgE from degranulation when these cells were incubated with 2S albumin or an IgE epitope (synthetic peptides). Here we provided the first experimental evidence supporting the fundamental role of amino acids with side chains containing acidic groups (mainly glutamic acid) in the formation of IgE epitopes of castor bean allergens. Worldwide, 10–20 million people suffer from pollen allergies [17], [18]. Vieths et al. [19] have indicated that between 50% and 93% of birch pollen allergic individuals develop hypersensitivity reactions towards certain foods (e.g., apples, carrots, hazelnut, celery), which is mediated by cross-reactive IgE antibodies primarily directed against Bet v 1, a major birch pollen allergen [18], [19]. This type of hypersensitivity was described as pollen-food syndrome (PFS) [18], [20]. The identification of aeroallergens and food allergens that cross-react with castor bean is an important step towards developing strategies for the prevention and treatment of allergies triggered by these components. The cross-reactivity between castor bean 2S albumin and allergens from shrimp, fish, wheat, soybean, peanut and corn causes concern because individuals who live in regions close to castor bean farms could become sensitive to these foods. The involvement of charged amino acids in Blo t 5, the most prevalent Blomia tropicalis allergen, with IgE binding was observed in another study [21]. These authors have demonstrated that four charged residues: Glu76, Asp81, Glu86, and Glu91 (peptide ELKRTDLNILERFNYE), localized to the region connecting the alpha helices 2 and 3, have been found to be involved in IgE binding. The pre-absorption of the serum of atopic individuals with glutamic acid decreased IgE binding to *R. communis* allergens (Table 3). We note that this amino acid also affected mast cell degranulation and histamine release, not only triggered by Ric c 1 and Ric c 3, but also triggered by other allergens that showed cross-reactivity with Ric c 1 and Ric c 3. There is continuing concern with the development of new drugs for the treatment of allergy and allergic rhinitis. A list of the medications used in allergic rhinitis, including their mechanisms of action and side effects, was presented by two other studies [22], [23]. The results presented in this manuscript support a new strategy for drug development based on IgE blocking (Machado et al., Brazilian patent 00320/2005) [24].

## Supporting Information

Figure S1 (A) Hypersensitivity reactions: a) Allergenic proteins are processed by antigen-presenting cells (APC) by the MHC class II pathway with subsequent increases in IgE production; b) IgE sensitizes mast cells by binding to FcεRI; c) A second exposure to the allergen activates these cells to degranulate and release vasoactive amines. (B) Blockage of IgE: a) Sensitized mast cells are incubated with glutamic or aspartic acid; b) These dicarboxylic amino acids bind to IgE-sensitized mast cells resulting in protection from mast cell degranulation.
(TIF)
