# Supplementary figures and images for: Identification of Critical Amino Acids in the IgE Epitopes of Ric c 1 and Ric c 3 and the Application of Glutamic Acid as an IgE Blocker
Source: PLoS One. 2011 Jun 27;6(6):e21455. doi: 10.1371/journal.pone.0021455 (PMC3124516; doi:10.1371/journal.pone.0021455)

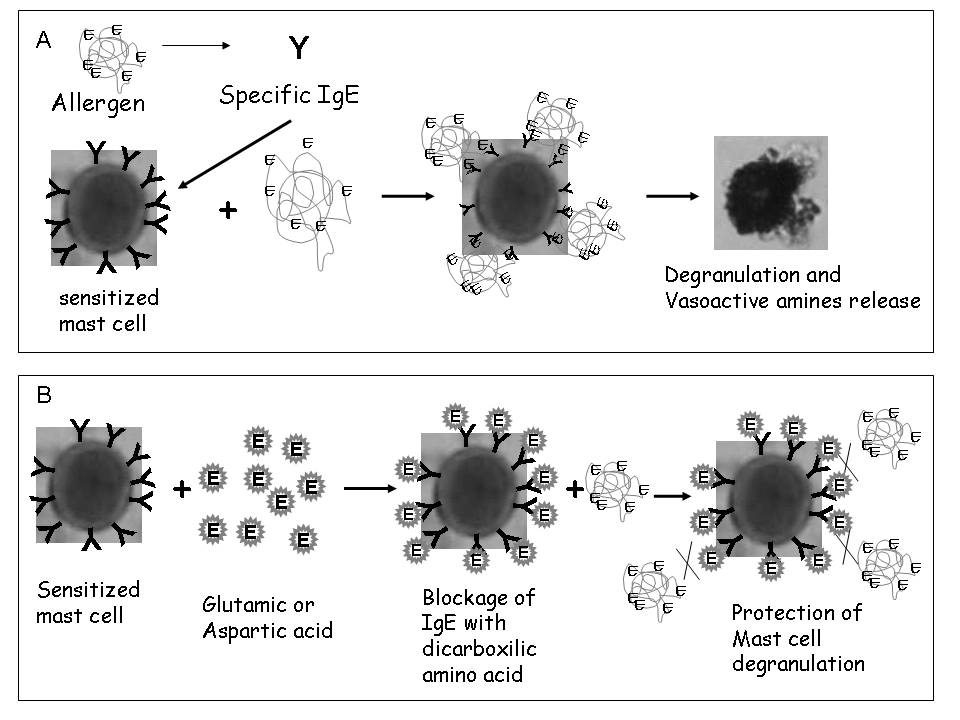

Supplement: Figure S1 — (A) Hypersensitivity reactions: a) Allergenic proteins are processed by antigen-presenting cells (APC) by the MHC class II pathway with subsequent increases in IgE production; b) IgE sensitizes mast cells by binding to FcεRI; c) A second exposure to the allergen activates these cells to degranulate and release vasoactive amines. (B) Blockage of IgE: a) Sensitized mast cells are incubated with glutamic or aspartic acid; b) These dicarboxylic amino acids bind to IgE-sensitized mast cells resulting in protection from mast cell degranulation. (TIF) [file pone.0021455.s001.tif]
